# Computational Analysis of Morphological Changes in *Lactiplantibacillus plantarum* Under Acidic Stress

**DOI:** 10.3390/microorganisms13030647

**Published:** 2025-03-12

**Authors:** Athira Venugopal, Doron Steinberg, Ora Moyal, Shira Yonassi, Noga Glaicher, Eliraz Gitelman, Moshe Shemesh, Moshe Amitay

**Affiliations:** 1 Biofilm Research Laboratory, Institute of Biomedical and Oral Research (IBOR), Faculty of Dental Medicine, The Hebrew University of Jerusalem, Jerusalem 9112102, Israel; athira.venugopal@mail.huji.ac.il; 2 Department of Food Quality and Safety, Institute for Postharvest Technology and Food Sciences, Agricultural Research Organization, Volcani Center, Rishon LeZion 50250, Israel; moshesh@volcani.agri.gov.il; 3 Department of Bioinformatics, Jerusalem College of Technology, Jerusalem 9372115, Israel; oramoyal770@gmail.com (O.M.); shirayonassi@gmail.com (S.Y.); nogagl901@gmail.com (N.G.); eliraz.git6@gmail.com (E.G.)

**Keywords:** *Lactiplantibacillus plantarum*, morphology, cell length, deep learning, object detection, image classification

## Abstract

Shape and size often define the characteristics of individual microorganisms. Hence, characterizing cell morphology using computational image analysis can aid in the accurate, quick, unbiased, and reliable identification of bacterial morphology. Modifications in the cell morphology of *Lactiplantibacillus plantarum* were determined in response to acidic stress, during the growth stage of the cells at a pH 3.5 compared to a pH of 6.5. Consequently, we developed a computational method to sort, detect, analyze, and measure bacterial size in a single-species culture. We applied a deep learning methodology composed of object detection followed by image classification to measure bacterial cell dimensions. The results of our computational analysis showed a significant change in cell morphology in response to alterations of the environmental pH. Specifically, we found that the bacteria existed as a long unseparated cell, with a dramatic increase in length of 41% at a low pH compared to the control. Bacterial width was not altered in the low pH compared to the control. Those changes could be attributed to modifications in membrane properties, such as increased cell membrane fluidity in acidic pH. The integration of deep learning and object detection techniques, with microbial microscopic imaging, is an advanced methodology for studying cellular structures that can be projected for use in other bacterial species or cells. These trained models and scripts can be applied to other microbes and cells.

## 1. Introduction

Bacteria have a unique capability to maintain precise cell morphology. These morphological characteristics are fundamental metric distinctions between bacteria of different species [1]. One of the main common categories of bacteria, based on their shape, is cocci, which may either remain single or attach to one another in groups (*Streptococci*). It may be assumed that coccoid forms were derived from rod-shaped organisms through evolutionary time. *Bacilli*, which are rod-shaped cells similar to cocci, either remain single or attach to other cells. Another type is the group that includes bacteria that are either helical-shaped or curved (comma-shaped), which can range from slightly curved to corkscrew-like spiraled [2].

The morphology of bacteria is sometimes taken as a constant value. However, variations in bacterial dimensions and shape may be indicative of the changes in physiological, virulent, or environmental factors [3]. In addition to this, morphological modifications may occur with growth rate in response to changes in temperature. Apparently, the energy consumed by bacteria also depends on their size [4]. Bacterial morphology influences survival, pathogenicity, motility, biofilm formation, and immune evasion. Cell shape enhances surface colonization and social motility [5]. In gastrointestinal pathogens, morphology aids mucus penetration and colonization [6]. Under stress, filamentation helps bacteria evade phagocytosis, persist in biofilms, and escape predation, highlighting morphology’s critical role in bacterial adaptation and pathogenesis [7].

Under certain environmental settings, such as biofilm growth conditions, low carbon bioavailability, and carbon–nitrogen imbalance, some bacteria embedded in the biofilm are elongated while maintaining constant width. Through elongation, biofilm bacteria strategically enlarge their nutrient collection surface without substantially changing the ratio of surface area to volume (SA/V). This shows that bacteria growing under restricted conditions may morphologically adapt to starvation by elongating [8].

The morphological traits of bacteria are controlled by diverse biochemical pathways and bacterial properties, such as cytoskeletal proteins, elasticity, fluidity of membranes, and osmotic pressure [9].

*Lactiplantibacillus plantarum* is a gram-positive, rod-shaped bacteria and they thrive in broad arrays of habitats due to their immense ecological and metabolic adaptability [10,11]. In addition, *L. plantarum* has gained the status of GRAS (generally regarded as safe) for their valuable application in the food and fermentation industry and as potential probiotic and postbiotic organisms [12]. The viability and stability of these bacteria can be affected by exposure to stressors such as thermal stress, cold storage, pH imbalance, osmotic shift, and other unstable conditions [13,14].

Morphological changes in response to stress are considered indicators of survival strategies in bacteria [15]. Cellular morphology exhibits dynamic plasticity, undergoing substantial structural modifications in response to environmental stressors, such as pH fluctuations, temperature variations, and nutrient availability [13,16]. Under acidic conditions, *Lactobacillus plantarum* undergoes elongation and alterations in cell wall integrity, facilitating enhanced survival and sustained metabolic activity [12]. Likewise, osmotic and thermal stress modulate its biofilm-forming capacity, a critical determinant of adhesion and persistence across diverse ecological niches [11]. Recent studies have further underscored the significance of encapsulation strategies in preserving *L. plantarum*’s structural integrity, particularly in probiotic and pharmaceutical formulations [13]. These morphological adaptations are integral to its functional resilience, influencing stress tolerance, biofilm development, and overall viability in industrial and clinical applications.

They do so by altering either their cell division pattern or the composition of the peptidoglycan membrane [16,17]. Morphological changes in *L. plantarum* can potentially affect its growth kinetics, viability, and adhesion abilities. Furthermore, these morphological alterations can also be advantageous by providing cross-protection against additional environmental changes [18].

As a common response to stress, many rod-shaped bacteria undergo filamentation, wherein the cell division process is hindered, leading to the chaining of the cells without separating from each other [15]. However, this type of adaptive morphotype is often overlooked by microscopic observations and lacks precise computational analysis. *L. plantarum* is often exposed to acidic environments as they release lactic acid into the medium during their growth. *L. plantarum* in acidic pH led to the display of phenotypic heterogeneity among the population, which enables it to improve its viability [18]. A V-shape structure of two bacteria is formed under acid stress [19,20]. This morphological change is associated with biofilm formation, quorum sensing, and is dependent on the LuxS/AI-2 pathway [20]. It has also been shown in our recent work that an incomplete cell division process leads to the phenomenon of V-shaped multicellular structuring [20]. However, the detailed quantitative characterization of the morphological changes associated with adaptation to acidic stress was not studied. Hence, computerizing the ultrastructural changes adopted by *L. plantarum* during acid stress is helpful for exploiting the benefits of this bacterium.

Bacterial morphology is an important microbial parameter that can provide vital information about the properties and ecological stages of bacteria. Several methods have been employed to measure bacterial size. Some examples include electron microscopy, coulter counter, flow cytometry epifluorescence microscopy, and transmission electron microscopy. Each of these methods has advantages and disadvantages.

The integration of deep learning into biological microscopy imaging has ushered in a new era of precision and efficiency in the study of cellular structures and processes [21]. With the advent of advanced neural network architectures, particularly convolutional neural networks (CNNs), deep learning algorithms have proven invaluable for the automated analysis and interpretation of microscopic images. This integration allows characterizations such as cell (or subcellular organelle) classification, segmentation, and detection to be performed with unprecedented accuracy and speed [22]. Size estimation involves the process of determining the dimensions or physical size of objects within an image. This task is fundamental in various applications ranging from fruit size [20,21] to cell dimension analysis [23,24].

One of the more advanced means used to measure bacterial cell dimensions is deep learning algorithms of computational vision. In this study, we used two computer vision methods: image classification and object detection. Image classification categorizes images into predefined classes based on their visual content. Object detection involves the identification and localization of objects within images. The goal is not only to recognize the types of objects present in an image but also to provide precise bounding box coordinates around each detected object. Both methods consist of training on datasets with labeled images to allow the algorithm parameters to capture intricate patterns and variations, allowing them to generalize new, unseen images.

The aim of this study was to develop a computational analysis of cell morphology, quantitatively characterizing the changes in *L. plantarum* morphology [19,20]. Our results provide a novel computational approach for detecting differences in morphological changes adopted by *L. plantarum* during its adaptation to acidic stress. These computational tools can be beneficial in studying other bacteria species or cells.

## 2. Materials and Methods

### 2.1. Bacterial Strains and Growth Conditions

*L. plantarum* 12422 (Lallemand, Blagnac, France) was used in this study for analysis and morphological characterization. For routine growth, *L. plantarum* was cultured in De Man, Rogosa, and Sharpe (MRS) medium (HiMedia Laboratories Pvt. Ltd., Maharashtra, India) at a pH of 6.5 or on MRS agar at 37 °C with 5% CO_2_, under non-shaking conditions. For acidic pH stress conditions, the MRS-6.5 medium was adjusted to a pH of 3.5 using 1M HCl. For each experiment, an overnight culture of *L. plantarum* cultured at a pH of 6.5 (OD_600_ approx. 2) was diluted to an OD_600_ of 0.1 using MRS-6.5 or MRS-3.5.

### 2.2. High-Resolution Scanning Electron Microscopy (HR-SEM)

HR-SEM (Electron Microscopy Sciences, Hatfield, PA, USA) was used to image *L. plantarum* when cultured in MRS-3.5 and MRS-6.5. The bacterial cells were collected after 24 h of growth, centrifuged (5000× *g*, 5 min, 4 °C), fixed in 4% glutaraldehyde (Electron Microscopy Sciences, Hatfield, PA, USA) in sterile water for 2 h, and washed twice with sterile water before letting them dry on a 0.5 × 0.5 cm glass slide. The samples were coated with iridium prior to imaging using a Cryo High-Resolution Scanning Electron Microscope (Apreo 2S, Thermo Fischer Scientific, Hatfield, PA, USA) at 5000× and 20,000× magnification. All samples were biologically and experimentally tested in triplicate.

### 2.3. Cell Dimensions Analysis from SEM Images

**Figure 1:**
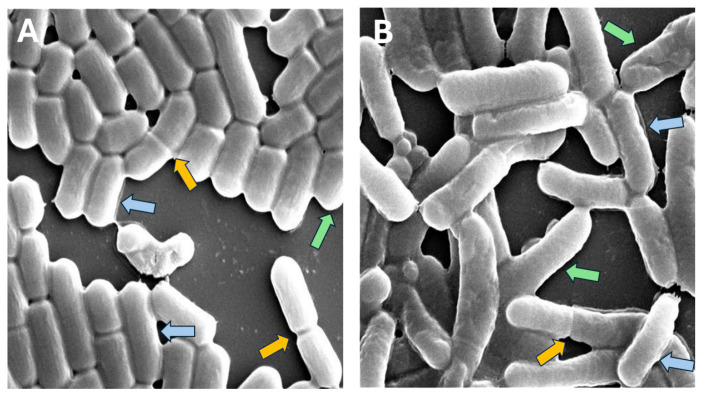
Examples of *L. plantarum* regular single cells (blue arrows), cells in the process of binary fission (yellow arrows), filamentous or unseparated cells (red arrows) and cells that are shortened by the image border or neighboring cells (green arrows): (**A**) control; (**B**) acidic condition.

Accurately measuring bacterial cell dimensions in microscopy images poses significant challenges, especially when dealing with complex and noisy visual data [25,26]. In our study, we encountered several obstacles in measuring the dimensions of rod-shaped cells (Figure 1).

1. Cells were shortened because of overlapping cells or partial cells near the borders of the image.
2. Cells showed binary fission, e.g., they were actually two cells, not a single cell.

**Figure 2:**
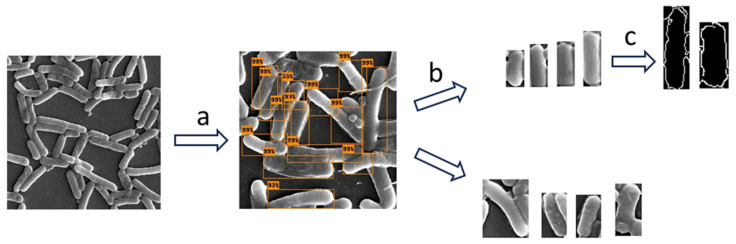
A diagram illustrating the workflow undertaken to measure the bacterial cell dimensions: (**a**) First, object detection was applied to isolate the cells from the SEM image. (**b**) Then, image classification was used to pick cell images with image dimensions that represented the dimension of the bacteria, and (**c**) in the final step, the Canny edge detection algorithm was applied to obtain more accurate measurements.

We did not use a specialized analysis software (such as SuperSegger) since we wanted to make sure that our methodology properly addressed these challenges [27]. The following methodology was applied to measure bacterial cell dimensions (depicted in Figure 2). We used object detection (identification and localization of objects within images) to identify and locate bacterial cells in the SEM images [28]. We then applied image classification (training of an algorithm to recognize patterns in images that distinguish one class from another) to select only the bacterial bounding boxes whose dimensions represented the dimensions of the bacterial cells. At this stage, we filtered out partial cells and cells in the stage of mitosis. In the following step, we applied the Canny algorithm to more accurately detect the edge of the bacterial cell in the filtered images and to calculate the width and length of the cell. We evaluated the computational results using manual measurements that were not included in the training. We used this methodology to compare the width and length of bacterial cells under the two conditions.

### 2.4. Image Classification: Acidic vs. Control

SEM images were obtained at a magnification of 5000. The initial SEM slides were modified from 1500 × 1000 pixels to 24 images of 250 × 250 pixels. Images of 20, 200, and 4400 magnitude were used for the training, validation, and testing sections, respectively. The image classification model was implemented using Keras version 2.15.0 [29]. Fine tuning was performed with the pretrained EfficientNetB0 architecture [30]. The following top layers were added: GlobalAveragePooling2D, a dense layer with 16 neurons, a dropout of 0.15, and an output layer of two neurons [28]. The images were pre-processed by resizing them to a uniform size of 128 × 128 pixels and normalizing pixel values. Data augmentation techniques, such as vertical and horizontal flipping, were applied. The model was trained (only the top layers) using an Adam optimizer [31] with a polynomial decay schedule of the learning rate, using an initial learning rate of 10^−3^. The categorical cross-entropy loss function was chosen as the optimization criterion.

### 2.5. Measurement of Bacterial Dimensions

#### 2.5.1. Step A: Object Detection

Object detection was conducted using images from an SEM magnification of 20,000. The image size was 500 × 500 pixels. We manually annotated the bounding boxes of only full-length bacterial cells (a single class). The dataset consisted of 135 images from both the acidic and control conditions (an equal quantity from each condition). The dataset was split into training validation and test sets with quantities of 50, 35, and 50. Data augmentation was performed by applying horizontal and vertical flips, zooming, and adjusting brightness and contrast.

Faster RCNN [32] architecture was applied with Resnet-50 as the backbone and Feature Pyramid Networks (FPN) of the TensorFlow Model Garden (TFM) module of TensorFlow 2. The performance of the trained model on the test set was average precision, mAP (0.5—0.95) of 0.71, and AP_50_ of 0.88 [33]. This object detection model was then used to obtain more than 25,000 images of separate bacterial cells for the next step of image classification.

#### 2.5.2. Step B—Image Classification

Image classification was conducted using images from the object detection output bounding boxes. We manually created two classes: (1) cell images whose image dimensions represent their cell dimensions; (2) cell images whose image dimensions did not represent their cell dimensions (sample images depicted in Figure 2, Step 3). Cells of both acidic and control conditions were included (an equal quantity from each condition). The dataset was randomly split into training, validation, and test sets. Each set consisted of 840, 240, and 120 images, respectively, and had an equal quantity from each class.

The image classification model was implemented using Keras v 2.15.0 [29]. Fine tuning of the pretrained EfficientNetV2 [30] architecture was performed. The following top layers were added: GlobalAveragePooling2D, dense layer with 16 neurons, a dropout of 0.15, and an output layer of 2 neurons. The images were preprocessed by resizing to a uniform size of 128 × 128 pixels and by normalizing pixel values. We applied data augmentation techniques, such as vertical and horizontal flipping.

The model was trained (only the top layers) using an Adam optimizer [31] with a polynomial decay schedule of learning rate, using an initial learning rate of 10^−3^. The training process involved 30 epochs, and the batch size was set to 16. The categorical cross-entropy loss function was chosen as the optimization criterion. The final accuracy of the trained model on the test set was 0.88. We then used this classifier to filter out, from more than 25,000 single-cell images, the images whose image dimensions represented their cell dimensions, and we obtained 1200 images. A manual examination was performed on these images, and we created a final dataset of 600 images (300 images from each condition: acidic and control). Additionally, the Canny edge detection algorithm [34] was applied to obtain a more accurate measurement of the width (using OpenCV Python package v 4.10) [30]. In order to further evaluate our results, we manually measured the length and width of 200 cells from this dataset.

All the codes and models are available in the following repository: https://github.com/OraMoyal26/bacteria_dimensions, accessed on 3 December 2024.

### 2.6. Growth Kinetic Studies

The bacterial growth profile of *L. plantarum* in MRS-6.5 and MRS-3.5 was evaluated by kinetic studies. An overnight culture of *L. plantarum* was diluted in 10 mL of either non-buffered MRS-6.5 or a mixture of MRS-3.5 medium with an initial OD_600nm_ of 0.1. A total of 200 µL of this culture was inoculated in a sterile tissue-grade transparent flat-bottomed 96-well microplate (Corning, Incorporated, Kennebunk, ME, USA), and bacterial growth was measured at an OD_600_ nm every 30 min for a period of 24 h in a Tecan M200 infinite microplate reader (Tecan trading AG, Mannesdorf, Switzerland). The experiment was performed in triplicate and expressed as mean ± standard deviation.

### 2.7. Laurdan Membrane Fluidity Assay

Changes in membrane fluidity associated with morphological changes were recorded using a fluorescent probe Laurdan, which intercalates with the peptidoglycan bilipid layer and emits a shift in the wavelength depending on the number of water molecules [35]. A total of 1 mL of OD 0.3 of *L. plantarum* suspension, cultured in a pH of 6.5 or a pH of 3.5 for 4 h, was centrifuged. Then, Laurdan (AnaSpec Inc., Fremont, CA, USA) was added to a final concentration of 10 µM, and the samples were incubated for 10 min at 37 °C. As for controls, unstained samples of a pH of 6.5 or a pH of 3.5 were also prepared. After incubation, the bacteria were washed four times with a mix of 1 mL PBS and 0.1% dimethylsulfoxide (DMSO). The fluorescence emitted from 200 µL of each sample, in triplicate and in a µ-clear black flat 96-well plate (Greiner Bio-One, Kremsmünster, Austria), was monitored in the M200 infinite plate reader (Tecan, Trading AG, Männedorf, Switzerland) with an excitation of 350 nm and an emission spectrum spanning from 400 nm to 600 nm at 30 °C. Membrane fluidity was measured using Laurdan generalized polarization (GP) values according to the formula GP = (RFI_440nm_ − RFI_490nm_)/(RFI_440nm_ + RFI_490nm_), as described in [35].

## 3. Results

### 3.1. Image Classification Method Shows That L. plantarum Shows Significant Differences in Morphology When Cultured in Acid Stress

**Figure 3:**
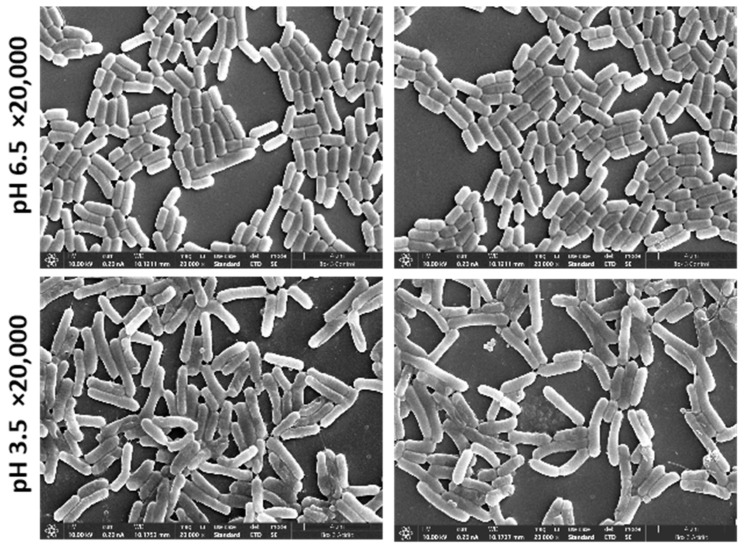
SEM images of *L. plantarum* of magnification ×20,000 that were used to train the algorithm. Upper panel, *L. plantarum* cultured in MRS-6.5; lower panel, cultures in MRS-3.5.

With the aim of examining the morphological changes in acidic conditions compared to the control, we performed SEM imaging of *L. plantarum* and applied the image classification method. We trained the algorithm to learn features and patterns in the images to accurately classify the two environmental states of control and acidic conditions (Figure 3 presents sample images from each state). To demonstrate that the two environmental conditions were significantly different, we used 20 images from the SEM for training (10 of each state). Additionally, the training images were taken from a single cultivation batch (Figure 3) and tested on the two other cultivation batches.

Our results demonstrated a nearly perfect prediction accuracy (number of correct predictions out of total number of predictions) of the unseen test set of 0.97 (the detailed confusion matrix is available in Table 1). The results showed that 97% of the 250 × 250 pixel images that were cut from the 5000 SEM magnification images in the acidic condition were different from the control. Bearing in mind that the training was performed on only 20 images, we can conclude that the morphological differences between the two conditions are consistent and significant.

### 3.2. Acidic Stress Leads to Slower Growth and Increase in Length of L. plantarum

**Figure 4:**
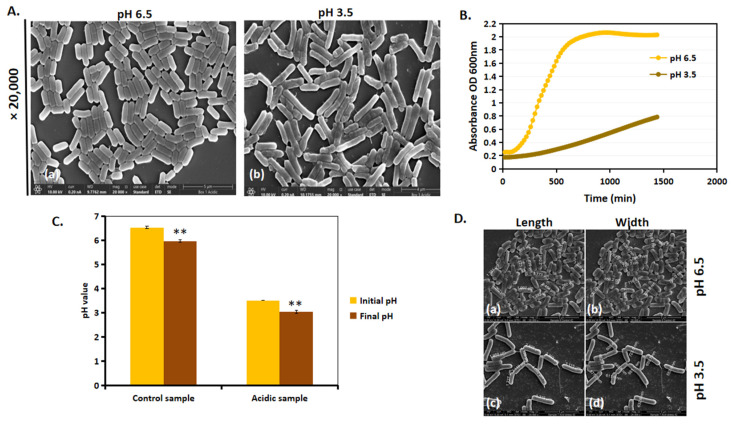
*L. plantarum* adapts to acidic stress by increasing their cell length. (**A**) SEM images of *L. plantarum* at ×20,000 magnification of cells at (**a**) a pH of 6.5 and (**b**) **a** pH of 3.5 after 24 h of incubation at 37 °C. (**B**) Growth kinetics of *L. plantarum* in a pH of 6.5 and a pH of 3.5 for a period of 24 h on a 96-well tissue grade polystyrene plate. (**C**) Initial and final pH values of the control and acid-stressed *L. plantarum* after 24 h of growth. (**D**) Manual measurements of length and width of *L. plantarum* at a pH of 6.5 (**a**,**b**) and a pH of 3.5 (**c**,**d**) from SEM images. ** represents *p* value < 0.05.

We next investigated whether the morphological differences between acidic and control conditions influenced *L. plantarum* growth. Growth patterns varied significantly between the group with a pH of 6.5 and that with a pH of 3.5, and these are observed in the SEM images (Figure 4A) and growth curve (Figure 4B). Previous findings [20] indicate that *L. plantarum* grown in a 50 mL Eppendorf tube at a pH of 3.5 exhibited a longer lag phase before initiating growth. However, after 24 h, the cells at a pH of 3.5 continued to grow exponentially, suggesting their ability to adapt and thrive under acidic conditions. A similar trend in the growth pattern was observed when cultured in a 96-well plate (Figure 4B). It is important to note that, since the pH of MRS-6.5 and MRS-3.5 was non-buffered, there was only a slight drop in pH; MRS-6.5 dropped to a pH of 6, and MRS-3.5 dropped to a pH of 3 (Figure 4C). SEM analysis (Figure 4D) revealed that, at a pH of 6.5, cells maintained a single rod-shaped morphology with no cell filamentation (Figure 4D(a,b)), whereas, at a pH of 3.5, cell length increased after 24 h (Figure 4D(c,d)). Cell filamentation, together with the increase in growth in a pH of 3.5, suggests that *L. plantarum* undergoes an adaptational change in acid stress.

### 3.3. Comparison of Bacterial Dimensions in Control vs. Acidic Conditions

We compared the length and width dimensions of the bacteria’s cells in the acidic and control environments. The accuracy of our computational approach was evaluated by comparing its measurements to the manual measurements. We obtained reasonable root mean square error (RMSE) deviations of 2.66 pixels for the length and 1.7 pixels for the width. This indicates a consistency between the computational and manual measurements.

Table 2 shows the cell dimensions based on the manual measurements comprised of 200 cells (100 cells from each experimental group). The median length of the acidic bacteria increased by 39%, whereas the width of the bacteria did not undergo a significant change. Moreover, the variation of the length nearly doubled in the acidic conditions.

**Figure 5:**
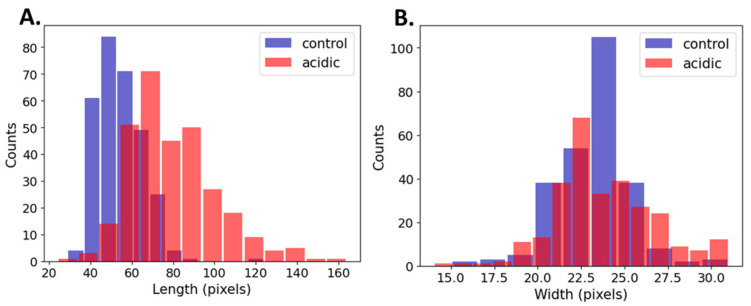
Histograms of (**A**) length (**left**) and (**B**) width (**right**) of control vs. acidic conditions.

Table 3 and Figure 5 show a summary of the cell’s dimensions in the acidic and control conditions based on a computational methodology comprising 600 cells (300 cells from each experimental group). The computational results were in agreement with those of the manual measurements (Table 3). The length of the median acidic bacteria increased by 41%, whereas the width of the bacteria underwent a significant change of only a single pixel. The larger sample size in the computational measurements increased the sensitivity to detect small effects; however, a single-pixel difference has no biological meaning. Additionally, the variation in length was shown to have nearly doubled in the acidic conditions.

### 3.4. Membrane Fluidity in L. plantarum Is Enhanced by Acidic Stress

**Figure 6:**
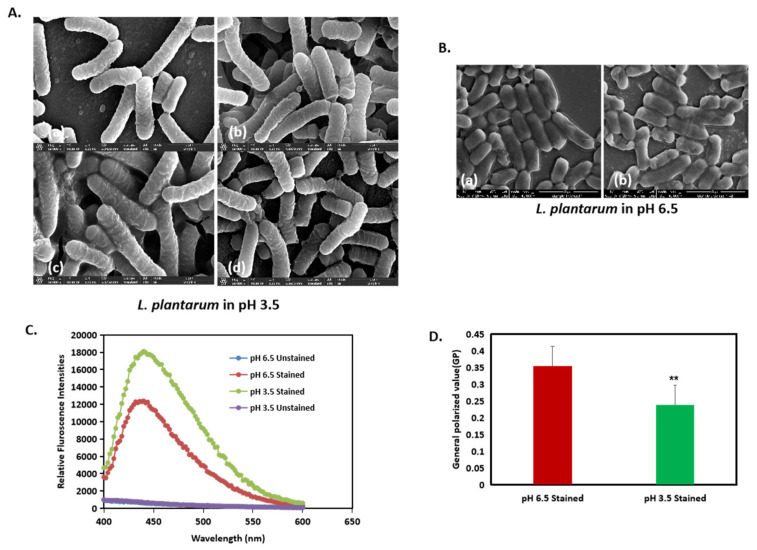
Acid stress induces changes in membrane fluidity in *L. plantarum*. (**A**(**a**–**d**)) and (**B**(**a**,**b**)): High-resolution SEM images of *L. plantarum* cells at a pH of 3.5 at ×50,000 magnification. (**C**) Membrane fluidity assay expressed as: relative fluorescence intensities and (**D**) generalized polarized (GP) value. ** represents *p* value < 0.05.

As it is apparent that a change in cell morphology occurred, we tested the fluidity of the membrane in the different pH conditions. Through visual observation, we observed that, at a pH of 3.5, the cells shared their outer membrane with the neighboring cells with no clear boundaries at the end of the cells (Figure 6A). However, at a pH of 6.5, the membrane between two cells could be clearly distinguished from each other (Figure 6B(a,b)). To further substantiate the findings, we performed the bacterial membrane fluidity assay by harvesting the bacteria after 24 h from both a pH of 6.5 and a pH of 3.5 and staining them with Laurdan stain, which is a fluorescent probe that intercalates to the region in the peptidoglycan membrane and displays an emission wavelength shift depending on the number of water molecules present between them. The relative fluorescence intensity, which measures the degree of membrane fluidity, was inversely proportional to the generalized polarized value (GP). Further, an increase in Laurdan staining indicates increased membrane fluidity. Our results showed that, at a pH of 3.5, the cells were more stained with the Laurdan stain compared to that of a pH of 6.5, indicating that there is an increase in membrane fluidity at a pH of 3.5 (Figure 6C). Additionally, the generalized polarized (GP) values (Figure 6D) showed that, at a pH of 3.5, the cells had a lower GP value, supporting the result of increased membrane fluidity.

## 4. Discussion

Bacteria are highly responsive to environmental changes, often undergoing morphological adaptations to enhance survival. In our recent study [20], we observed that *Lactiplantibacillus plantarum* exhibits a distinct V-shape morphology. This V-shaped structuring was characterized through manual observation, where the cells appeared to align in a “V” configuration of 4–8 cells attached to a common structural area [19,20], which is associated with acid stress. Previous studies have also reported other morphological changes in *L. plantarum* using basic visual inspection [16,36,37]. However, while visual observation can reveal general trends, it is challenging to quantify more intricate cell features, such as filamentation or surface area-to-volume ratios, that are not easily detectable with the naked eye. In our study, we demonstrated by computational analysis that the morphology of *L. plantarum* differs significantly in an acidic environment compared to control conditions. Remarkably, we used an image classification model, which was trained with a few images (20 images) and resulted in near-perfect accuracy in predicting the growth environment depicted in the images (Figure 3). This shows that the morphological differences between the two conditions are significant and easily distinguishable even with minimal training data.

In the next computational analysis step, we focused on two key features: growth rate and cell dimension analysis. Our findings from the growth kinetics indicated that there was a slow increase in growth of *L. plantarum* when cultured in a pH of 3.5. However, after 24 h of growth, the cells continued to grow exponentially when compared to that of cells cultured in a pH of 6.5 (Figure 4B). As the growth medium was not buffered, we monitored the potential pH decline after 24 h of growth. Our observations revealed only a slight decrease in the pH values for both *L. plantarum* control and acid-stressed samples (Figure 4C). Additionally, we noted that the initial pH of the growth media in both conditions remained stable for an extended period. A similar trend in growth profile was also observed previously in [20], when cells were cultured in bigger volumes of 50 mL Eppendorf tubes. Manual measurements of the cell length and width from the SEM images showed an increase in the length of the cells in a pH of 3.5 than those in a pH of 6.5 (Figure 4D). Further, a computational approach was utilized to accurately assess the size differences of *L. plantarum* under acidic stress compared to standard growth conditions (Table 3). Through this approach, we sampled two large populations of bacteria, a control group and a test group. However, to minimize false positives and negatives, the algorithm was trained to exclude images of bacteria that did not exhibit clear and well-defined geometric features. By using modified algorithms tailored to specifically characterize and measure dimensional features, we obtained more precise information on changes in cell dimensions.

We developed a custom-fit computational methodology for measuring the width and length of rod-shaped bacteria, with the primary goal of comparing cell dimensions under two conditions. This methodology involved object detection followed by image classification. By utilizing computerized imaging, we achieved the quick, accurate, and reliable detection of cell characteristics within a culture. By applying this methodology to sample and measure cell dimensions, we achieved results that closely aligned with manual measurements, showing a size increase of 41% vs. 39%, respectively (Table 2 and Table 3). Importantly, the same computational procedure, with identical parameter settings, was applied to each condition, ensuring that any bias or discrepancies were consistently reflected across both conditions.

Another key finding of this study was that the membrane fluidity significantly increased under the acidic pH compared to that of the control (Figure 6). This increase in fluidity facilitated morphological changes (Figure 6C), such as the long unseparated cell filaments forming the V-shaped structures with the shared outer membrane [20]. Further, Laurdan staining showed a lower generalized polarized (GP) value, indicating significant changes in the membrane fluidity and allowing the *L. plantarum* cells to adjust their cell dimensions in terms of cell length (Figure 6D). This increase in cell length was recorded in our computational analysis (Figure 5). It is plausible that membrane properties, which are integral to maintaining bacterial shape, undergo modifications during morphological adaptations. Furthermore, other types of bacteria are also sensitive to pH, as they change morphological structure in response to environmental pH. Acidic conditions were found to influence the length at which *Escherichia coli* initiated the division process [38]. The change in length of bacteria in the different pH environments, with very little change in bacteria width, indicated a specific targeted effect on cell morphology [39]. Moreover, the coordinated regulation of cell size provided flexibility, enabling bacteria to adjust their dimensions to meet changing environmental demands and to enhance survival under new conditions [40]. This may be associated with the formation of the V-shaped pattern of these bacteria at a low pH [20].

Finally, the computational methodology used does not require extensive or overly complex dataset labeling efforts, as both object detection and classification tasks are straightforward, making it relatively easy to implement in other studies. Most studies on size determination use object detection to isolate objects within images, with some employing semantic segmentation to define precise boundaries for the accurate measurement of size or diameter [41,42,43]. In this study, we opted for image classification to specifically address the need to classify and exclude partial, overlapping, and dividing cells. All the code, trained models, and datasets (the raw data, the object detection, and the image classification training datasets) of this study are publicly available in the GitHub repository and can be used for the dimensional analysis of other rod-shaped cells. The methodologies developed in this study offer a novel approach for detecting and quantifying bacterial morphology in cell cultures. This methodology can be extended to analyze other bacterial species or cell types under varying environmental conditions.

## 5. Conclusions

We developed a computational methodology for the accurate measurement of *L. plantarum* dimensions under varying environmental pH conditions, achieving results comparable to manual measurements. The advantage over manual measurements is the ability to screen numerous images in a short period of time. Additionally, by integrating object detection and image classification, the applied method can be targeted to specific bacterial morphology, effectively filtering out partial and dividing cells from the population, ensuring consistent and unbiased analysis across conditions. This straightforward implementation, requiring minimal dataset labeling, offers a reliable tool for bacterial morphological analysis that is applicable to various cell types and environmental conditions. The datasets, code, and trained models used for the study are publicly available for broader applications in microbial research.

## Figures and Tables

**Table 1:** Confusion matrix of the image classification classifier: control vs. acidic.

Test Samples	Predicted Control	Predicted Acidic
Actual control	2182	18
Actual acidic	116	2084

**Table 2:** Comparison of cell dimensions in the control vs. acidic conditions (manual measurements).

	Average	Median	SD	*p*-Value ^b^
Length Control	52.41	51	10.87	<<0.05
Length Acidic	75.89	71	21.17	
Width Control	23.81	24	1.97	0.34
Width Acidic	23.7	23	2.67	

The measurements, conducted manually, were recorded in pixel units; the dataset comprised 200 cells (100 cells from each group). ^b^ We applied the Mann–Whitney U test. This choice was made due to a non-normal distribution observed in the data, as confirmed by Shapiro–Wilk and D’Agostino–Pearson tests. A statistically significant change was seen only in the cell’s length.

**Table 3:** Comparison of cell dimensions in control vs. acidic conditions (computational measurements ^a^).

	Average	Median	SD	*p*-Value ^b^
Length Control	54.1	53	11	<<0.05
Length Acidic	79.42	75	21.14	
Width Control	23.01	23	2.07	7.0 × 10^−3^
Width Acidic	23.84	24	2.88	

^a^ The measurements, conducted computationally, were recorded in pixel units; the dataset comprised 600 cells (300 in each group). ^b^ We applied the Mann–Whitney U test. This choice was made due to a non-normal distribution observed in the data, as confirmed by Shapiro–Wilk and D’Agostino–Pearson tests. A statistically significant change was seen only in the length of the cells.

## Data Availability

Raw data are available upon reasonable request. GitHub repository also contains the link to the raw images.
